# Lysophosphatidic Acid Receptor 5 Contributes to Imiquimod-Induced Psoriasis-Like Lesions through NLRP3 Inflammasome Activation in Macrophages

**DOI:** 10.3390/cells9081753

**Published:** 2020-07-22

**Authors:** Bhakta Prasad Gaire, Chi-Ho Lee, Wondong Kim, Arjun Sapkota, Do Yup Lee, Ji Woong Choi

**Affiliations:** 1 College of Pharmacy and Gachon Institute of Pharmaceutical Sciences, Gachon University, Incheon 21936, Korea; samarpanbp@gmail.com (B.P.G.); lch7835@nate.com (C.-H.L.); wdongkim@gmail.com (W.K.); sapkotaa07@gmail.com (A.S.); 2 Department of Agricultural Biotechnology, Center for Food and Bioconvergence, Research Institute for Agricultural and Life Sciences, Seoul National University, Seoul 08826, Korea; rome73@snu.ac.kr

**Keywords:** lysophosphatidic acid receptor 5, TCLPA5, psoriasis, NLRP3 inflammasome, macrophages

## Abstract

The pathogenesis of psoriasis, an immune-mediated chronic skin barrier disease, is not fully understood yet. Here, we identified lysophosphatidic acid (LPA) receptor 5 (LPA_5_)-mediated signaling as a novel pathogenic factor in psoriasis using an imiquimod-induced psoriasis mouse model. Amounts of most LPA species were markedly elevated in injured skin of psoriasis mice, along with LPA_5_ upregulation in injured skin. Suppressing the activity of LPA_5_ with TCLPA5, a selective LPA_5_ antagonist, improved psoriasis symptoms, including ear thickening, skin erythema, and skin scaling in imiquimod-challenged mice. TCLPA5 administration attenuated dermal infiltration of macrophages that were found as the major cell type for LPA_5_ upregulation in psoriasis lesions. Notably, TCLPA5 administration attenuated the upregulation of macrophage NLRP3 in injured skin of mice with imiquimod-induced psoriasis. This critical role of LPA_5_ in macrophage NLRP3 was further addressed using lipopolysaccharide-primed bone marrow-derived macrophages. LPA exposure activated NLRP3 inflammasome in lipopolysaccharide-primed cells, which was evidenced by NLRP3 upregulation, caspase-1 activation, and IL-1β maturation/secretion. This LPA-driven NLRP3 inflammasome activation in lipopolysaccharide-primed cells was significantly attenuated upon LPA_5_ knockdown. Overall, our findings establish a pathogenic role of LPA_5_ in psoriasis along with an underlying mechanism, further suggesting LPA_5_ antagonism as a potential strategy to treat psoriasis.

## 1. Introduction

Psoriasis is a chronic and immune-mediated skin disease that is commonly characterized by thick, red, and itchy areas of skin. Epidermal acanthosis, hyperkeratosis, activation and infiltration of immune cells, and increased production of proinflammatory mediators from infiltrated immune cells are cardinal features of psoriasis [1,2]. Although the pathogenesis of psoriasis remains unclear, it is associated with complex etiological factors primarily driven by aberrant immune responses in the skin [3,4]. Among these, infiltration and activation of macrophages have been proven to be critical pathogenic events in psoriasis [5,6]. Therefore, managing inflammatory responses, particularly recruitment and activation of macrophages, could be a potential therapeutic strategy to treat psoriasis.

Lysophosphatidic acid (LPA), a bioactive lysophospholipid, is present throughout the body, including the skin. LPA regulates inflammatory responses in various diseases through its six LPA receptors (LPA_1–6_) [7,8]. LPA signaling regulates not only physiological skin functions, such as skin protection, metabolism, and sensation, but also pathological skin functions, including pruritus, skin tumors, scleroderma, and skin inflammation [9]. These diverse roles of LPA in the skin may indicate that LPA could actively participate in the pathogenesis of psoriasis. Indeed, amounts of LPA have been found to be significantly elevated in the plasma of human patients with psoriasis [10]. Moreover, its receptors might play a critical role in the pathogenesis of psoriasis. BMS-986202, a selective LPA_1_ antagonist, has undergone a Phase I clinical trial for psoriasis (ClinicalTrials.gov ID: NCT02763969) [11]. However, it remains unknown whether other LPA receptor subtypes are also involved in the pathogenesis of psoriasis.

LPA_5_ could be an additional LPA receptor subtype that might play a critical role in the pathogenesis of psoriasis. LPA_5_ is highly expressed in small intestine and moderately expressed in various tissues of mouse, including skin, spleen, and stomach [7,12,13]. It is highly expressed on cells associated with the immune system, such as lymphocytes and mast cells [12,14]. A recent transcriptomic study has also revealed that LPA_5_ is highly expressed on macrophages [15]. Furthermore, it has been shown that LPA_5_ is highly expressed in dorsal root ganglion and its signaling is involved in LPA-induced itch in mice [13,16]. LPA_5_ was reported to be highly expressed in normal human epidermal keratinocytes [17]. It has also been suggested as a putative regulator of keratinocyte differentiation and skin barrier function [17], both of which are regarded as important events in psoriasis [4]. However, whether LPA_5_ contributes to tissue injury of psoriasis remains unclear.

In the current study, we investigated the role of LPA_5_ in the pathogenesis of psoriasis. We employed an imiquimod (IMQ)-induced mouse psoriasis model [18]. We determined amounts of different LPA species in both injured skin and plasma of psoriasis mice by liquid chromatography-mass spectrometry (LC/MS) and LPA_5_ upregulation in psoriasis lesions by qRT-PCR and immunofluorescence. To address roles of LPA_5_ in psoriasis, we employed a specific LPA_5_ antagonist, TCLPA5 [19]. To address how LPA_5_ signaling might contribute to skin injury in psoriasis, we determined its role in macrophages, particularly in their NLRP3 inflammasome activation using in vivo psoriasis mice and in vitro lipopolysaccharide (LPS)-primed bone marrow-derived macrophages (BMDMs). Our results suggest that LPA_5_ is a novel pathogenic factor in psoriasis, along with its regulatory mechanisms in macrophage NLRP3 inflammasome activation.

## 2. Materials and Methods

### 2.1. Study Design and TCLPA5 Administration

All animal handling and experimental procedures were approved by the Institutional Care and Use Committee at Gachon University (approved animal protocol number: LCDI-2017-0083). Following a week of laboratory acclimatization of male BALB/c mice (6 weeks old, Orient Bio, Gyeonggi-do, Korea), dorsal back hair was removed using a hair-removal cream. Two days later, mice were randomly divided into sham, IMQ, and IMQ+TCLPA5 (Tocris Bioscience, Bristol, UK) administration groups. To induce psoriasis-like symptoms, 5% IMQ cream (Aldara, 62.5 mg) was topically applied to both dorsal shaved skin (about 3 × 4 cm^2^ area) and the right ear for six consecutive days. For the sham group, equal volumes of Vaseline were used. To suppress LPA_5_ activity, we used TCLPA5. It was first reported by Sanofi Aventis as a selective antagonist for LPA_5_ (IC_50_ = 0.8 μM in RH7777 cells overexpressing human LPA_5_) and confirmed to inhibit LPA-mediated human platelet aggregation with an IC_50_ value of 2.2 μM [19]. For the TCLPA5 administration group, TCLPA5 (0.5, 2, and 5 mg/kg, dissolved in 1:1 Cremophor EL:Ethanol and diluted in water) was intraperitoneally injected just before IMQ application for six consecutive days. For the IMQ group, equal volumes of vehicle were injected.

### 2.2. Psoriasis Area and Severity Index (PASI) Evaluation

The severity of psoriasis was determined daily for seven days by evaluating psoriasis area and severity index (PASI) scores, including skin scaling, erythema, and ear thickness, as described previously [20]. Skin erythema and scaling score ranged from 0–4 (0, no symptoms; 1, mild; 2, moderate; 3, severe; 4, very severe). Ear thickness was measured using a Vernier Caliper (Mitutoyo, Japan).

### 2.3. Tissue Preparation

On day 7, mice were sacrificed with CO_2_ inhalation. Pieces of skin tissue were harvested for biochemical or histochemical analysis. Skin tissues for histochemical analysis were fixed overnight in 4% paraformaldehyde (PFA), embedded in paraffin, and cut (3 µm) using a microtome (HM355S Microm, Thermo Fisher Scientific, Waltham, MA, USA). For biochemical analysis, skin tissues were preserved in liquid nitrogen and stored at −80 °C until used.

### 2.4. LC/MS Analysis

Skin samples (150 mg) and blood plasma samples (50 µL) from sham or IMQ-treated mice were extracted using the Folch method with minor modification [21,22]. Extracts were concentrated to complete dryness and reconstituted with 70% acetonitrile. Reconstituents were separated with a BEH C18 column (Waters Corporation, Milford, MA, USA). LC/MS analysis was conducted with an Ultimate-3000 UPLC system coupled to an Orbitrap mass spectrometry analyzer (Thermo Fisher Scientific). LPA species were identified against LipidBlast library [23].

### 2.5. H&E Staining

Paraffin-embedded skin sections were immersed in xylene (10 min × 3) and rehydrated with descending grades of ethanol (100%, 90%, 70%, and 50%) and water. For H&E staining, sections were stained with hematoxylin solution, washed several times with water, and incubated with eosin solution. Sections were then washed with water, dehydrated with ascending grades of ethanol, cleared in xylene, and cover-slipped. Stained sections were photographed using a bright field microscope (BX53T, Olympus, Japan). Representative images were prepared using Adobe Photoshop Elements 8. Skin thickness was manually measured with a ruler in a blind fashion for obtained images of stained skin sections and converted into µm based on a scale bar in the image.

### 2.6. Immunofluorescence

Skin sections were fixed with 4% PFA, exposed to antigen retrieval buffer (0.01 M sodium citrate) at 90–100 °C, blocked with 1% fetal bovine serum (FBS), and incubated with anti-F4/80 rat monoclonal antibody (1:100, Abcam, Cambridge, UK), anti-LPA_5_ rabbit polyclonal antibody (1:100, LifeSpan BioScience, Seattle, WA), or anti-NLRP3 mouse monoclonal antibody (1:200, AdipoGen Life Sciences, San Diego, CA, USA) overnight at 4 °C followed by labeling with a secondary antibody conjugated with AF488 or Cy3 (1:1000, Jackson ImmunoResearch, West Grove, PA, USA). Sections were counterstained with DAPI and mounted using VECTASHIELD (Vector Laboratories, Burlingame, CA, USA). For double immunofluorescence labeling, sections were co-labelled with antibodies against F4/80 and LPA_5_ or F4/80 and NLRP3 overnight at 4 °C followed by labeling with a secondary antibody conjugated with AF488 or Cy3. For image preparation, labeled sections were photographed using a confocal microscope (Eclipse A1 Plus, Nikon, Japan). The number of immunopositive cells for a mouse was obtained by calculating the mean value from three images (200 µm × 200 µm) in a blind fashion.

### 2.7. qRT-PCR and Semi-Quantitative PCR Analyses

Skin tissues were homogenized to extract total RNA using RNAiso plus (Takara, Kusatsu, Japan). StepOnePlusTM qRT-PCR system (Applied Biosystems, Foster City, CA, USA) and FG Power SYBR Green PCR master mix (Life Technologies, Carlsbad, CA, USA) were used for qRT-PCR analysis. Expression levels of each LPA receptor were quantified using the 2^−ΔΔCT^ method relative to 18S. To determine expression levels of pro-inflammatory cytokines (IL-1β, IL-17, and IL-23), semi-quantitative PCR was performed on a SimpliAmp Thermal cycler (Applied Biosystems) with AccuPower^®^ Taq polymerase (Bioneer, Daejeon, Korea). Image J software (National Institute of Mental Health, Bethesda, MD, USA) was used to quantify specific PCR products. The following primer sets were used: LPA_1_ For: GCAGCACACATCCAGCAATA Rev: GTTCTGGACCCAGGAGGAAT, LPA_2_ For: TCAGCCTAGTCAAGACGGTTG Rev: CATCTCGGCAGGAATATACCAC, LPA_3_ For: ACACCAGTGGCTCCATCAG Rev: GTTCATGACGGAGTTGAGCAG, LPA_4_ For: AGGCATGAGCACATTCTCTC Rev: CAACCTGGGTCTGAGACTTG, LPA_5_ For: AGGAAGAGCAACCGATCACAG Rev: ACCACCATATGCAAACGATGTG, LPA_6_ For: TGTGAGATGGGCTGTCTCTG Rev: ACTGGGTTGAAGCCTTCCTT, IL-1β For: GCCTTGGGCCTCAAAGGAAAGAATC Rev: GGAAGACACAGATTCCATGGTGAAG, IL-17 For: GCTCCAGAAGGCCCTCAGACT Rev: CCAGCTTTCCCTCCGCATTGA, IL-23 For: CCCACAAGGACTCAAGGACAA Rev: AGTAGGGAGGTGTGAAGTTGC, and 18S For: CCATCCAATCGGTAGTAGCG Rev: GTAACCCGTTGAACCCCATT.

### 2.8. Mouse Bone Marrow-Derived Macrophage (BMDM) Culture

Bone marrow cells were isolated from leg bones of male ICR mice (8 weeks old, Orient Co. Ltd., Gyeonggi-do, Korea) and differentiated into BMDM cells for three days in α-MEM supplemented with 10% heat-inactivated FBS, 1% penicillin/streptomycin, and 30 ng/mL recombinant mouse macrophage colony stimulating factor at 37 °C in a 5% CO_2_ incubator as described previously [24].

To activate NLRP3 inflammasome in cells, BMDM cells (5 × 10^6^ cells/well in a 6-well plate) were starved overnight, primed with LPS (500 ng/mL, Sigma-Aldrich, St. Louis, MO, USA) for 4 h, and exposed to LPA (Avanti Polar Lipids, Birmingham, AL, USA) for an additional 1 h. To determine effects of LPA itself, serum-starved cells were exposed to LPA for 4 h. As the vehicle, 0.1% fatty acid-free bovine serum albumin (FAFBSA, Sigma-Aldrich) was used.

Alternatively, BMDM cells were transiently transfected with LPA_5_ siRNA or control siRNA with Lipofectamine^®^ RNAiMAX reagent (Life Technologies) in serum- and antibiotics-free α-MEM. After 6 h, cells were recovered by incubation in α-MEM containing serum and antibiotics for 2 days. These cells were serum starved overnight, primed with LPS, and exposed to LPA. Knockdown efficiency of LPA_5_ siRNA was confirmed by Western blot analysis.

### 2.9. Western Blot

Protein samples obtained from BMDM cells were separated by SDS-PAGE and transferred to PVDF membranes (Merck Millipore, Burlington, MA, USA). These membranes were blocked with 5% skim milk and incubated overnight with primary antibodies against LPA_5_ (1:1000, LifeSpan BioScience, Seattle, WA, USA), NLRP3 (1:1000), procaspase 1 (1:1000, Abcam), caspase-1 (1:1000, AdipoGen Life Sciences), pro IL-1β (1:1000, Cell Signaling Technology, Danvers, MA, USA), mature IL-1β (1:1000, Abcam), and β-actin (1:10,000, Bethyl Laboratories, Montgomery, TX, USA) followed by incubation with HRP-conjugated secondary antibodies (1:10,000, Santa Cruz Biotechnology, Dallas, TX, USA). Protein bands were visualized using an enhanced chemiluminescence detection kit (Donginbiotech Co., Seoul, South Korea). Image J software was used to quantify target protein bands.

### 2.10. ELISA

Conditioned medium was collected from BMDMs, concentrated by VIVASPIN 500 (Sartorius, Goettingen, Germany), and processed for ELISA to measure concentrations of IL-1β according to the manufacturer’s protocol (R&D systems, Minneapolis, MN, USA).

### 2.11. Statistical Analysis

Data are presented as mean ± S.E.M.. Statistical analyses were performed using GraphPad Prism 7 (GraphPad Software Inc., San Diego, CA, USA). Statistical differences between two groups were evaluated with Student’s t-test. Statistical differences among multiple groups were evaluated with one-way ANOVA or two-way ANOVA followed by Newman–Keuls post-test. Statistical significance was set at *p* < 0.05.

## 3. Results

### 3.1. Activation of LPA_5_ Signaling Contributes to Skin Injury in Mice with IMQ-Induced Psoriasis

To test whether the amount of LPA in psoriasis might be increased in mice as it was elevated in the plasma of psoriasis patients [10], we treated BALB/c mice with IMQ and profiled LPA species using LC/MS analysis. Amounts of more than half of the LPA species were significantly increased in injured skin (Table 1) of IMQ-treated group compared to sham group. In plasma, amounts of a few LPA species were significantly elevated (Table 1). Such quantitative increase of LPA species was pronounced in injured skin.

**Figure 1:**
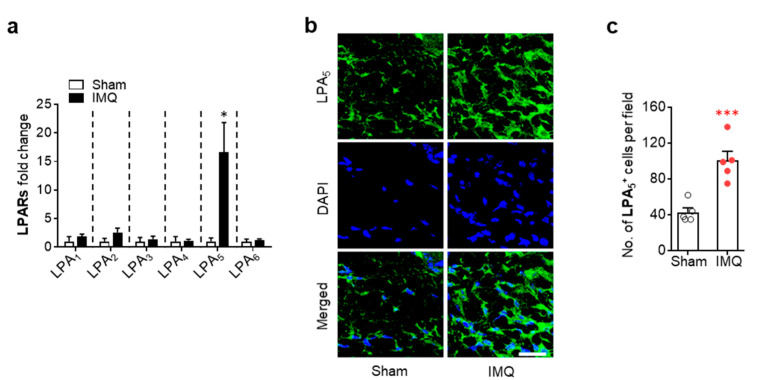
LPA_5_ expression is upregulated in psoriasis lesions of IMQ-treated mice. (**a**) mRNA expression levels of LPA receptor subtypes in skin samples from sham and IMQ-treated mice were analyzed at 7 days after IMQ treatment using qRT-PCR analysis. n = 4 for sham and n = 5 for IMQ. Two-tailed t-test. * *p* < 0.05 vs. sham. (**b**) Representative photographs of LPA_5_-labelled skin sections were taken from the dermis of each group. DAPI was used for nuclear staining. Scale bar = 20 µm. (**c**) Quantification of the number of LPA_5_-immunopositive cells per field (200 µm × 200 µm) was manually performed. n = 5 per group. Two-tailed t-test. *** *p* < 0.001 vs. sham.

We next determined whether LPA_5_ expression could be altered in injured skin of IMQ-treated mouse by qRT-PCR analysis. Expression levels of LPA_5_ mRNA were dramatically increased in psoriasis lesions, whereas mRNA expression levels of other LPA receptor subtypes were not significantly altered (Figure 1a). LPA_5_ upregulation was also observed at protein levels as evidenced by increase in the number of LPA_5_-immunopositive cells in the dermis of psoriasis lesion (Figure 1b,c). These results indicate that LPA_5_-mediated LPA signaling could be a critical factor in the pathogenesis of psoriasis.

**Figure 2:**
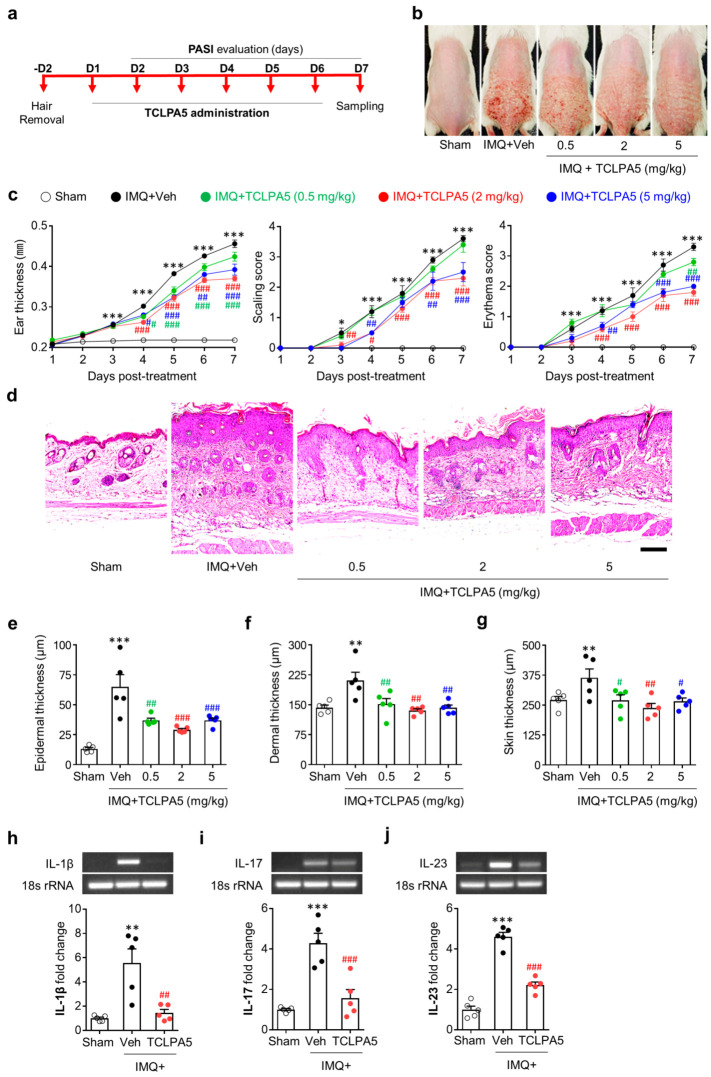
LPA_5_ antagonism reduces IMQ-induced psoriasis-like symptoms in mice. (**a**) Schematic illustration of experimental procedures performed in this study. (**b**) Representative photographs of skin on the back were taken from mice of each group at day 7 as described in ‘a’. (**c**) Measurements of ear thickness, skin scaling, and skin erythema were performed daily to quantify PASI scores. n = 5 per group. Two-way ANOVA and Newman–Keuls test. * *p* < 0.05 and *** *p* < 0.001 vs. sham; ^#^ *p* < 0.05, ^##^ *p* < 0.01, and ^###^ *p* < 0.001 vs. IMQ-treated group (IMQ+Veh). (**d**) Representative photographs of Hematoxylin and Eosin-stained skin samples were taken from mice of each group at day 7 after IMQ application. Scale bar = 100 µm. (**e**–**g**) Quantification of epidermal thickness (**e**), dermal thickness (**f**), and total skin thickness (**g**) was performed by measuring thickness of each skin layer. n = 5 per group. One-way ANOVA and Newman–Keuls test. ** *p* < 0.01 and *** *p* < 0.001 vs. sham; ^#^ *p* < 0.05, ^##^ *p* < 0.01, and ^###^ *p* < 0.001 vs. IMQ-treated group (IMQ+Veh). (**h**–**j**) Effects of TCLPA5 (2 mg/kg) on mRNA expression levels of IL-1β (**h**), IL-17 (**i**), and IL-23 (**j**) in skin from IMQ-treated mice were analyzed at 7 days using semi-quantitative PCR analysis. Representative gel (**h**–**j**, upper panels) and quantification of results (**h**–**j**, lower panels). n = 5 per group. One-way ANOVA and Newman-Keuls test. ** *p* < 0.01 and *** *p* < 0.001 vs. sham; ^##^ *p* < 0.01 and ^###^ *p* < 0.001 vs. IMQ-treated group (IMQ + Veh).

To address the pathogenic role of LPA_5_ in psoriasis, we administered TCLPA5 to IMQ-treated mice for six consecutive days (Figure 2a). Topical application of IMQ dramatically increased PASI scores, including skin erythema, scaling, and ear thickness (Figure 2b,c). Conversely, administration of TCLPA5 at daily dosage of 2 mg/kg or 5 mg/kg remarkably attenuated these PASI scores (Figure 2b,c), indicating a pathogenic role of LPA_5_ in psoriasis. At daily dosage of 0.5 mg/kg, TCLPA5 administration also significantly decreased ear thickness (Figure 2c). However, it did not affect skin scaling at all time points and attenuated skin erythema at a single time point (day 7) (Figure 2c).

To further address the pathogenic role of LPA_5_ in psoriasis, we determined whether TCLPA5 administration could attenuate psoriasis-induced skin thickening using hematoxylin and eosin (H&E)-stained skin tissue sections. TCLPA5 administration significantly decreased IMQ-induced skin thickening as evidenced by its attenuation of IMQ-induced increase in dermal, epidermal, and total skin (epidermis + dermis + hypodermis) thicknesses (Figure 2d–g). Because the effects of TCLPA5 on PASI parameters were more pronounced at 2 mg/kg (Figure 2b–g), this dosage was used for further in vivo experiments.

We also determined mRNA expression levels of IL-1β, IL-17, and IL-23, all of which are major cytokines associated with psoriasis [4,25,26,27], by semi-quantitative PCR analysis. TCLPA5 administration significantly attenuated IMQ-induced upregulation of these cytokines (Figure 2h–j).

### 3.2. LPA_5_ Regulates Macrophage Infiltration in the Dermis of Mice with IMQ-Induced Psoriasis

**Figure 3:**
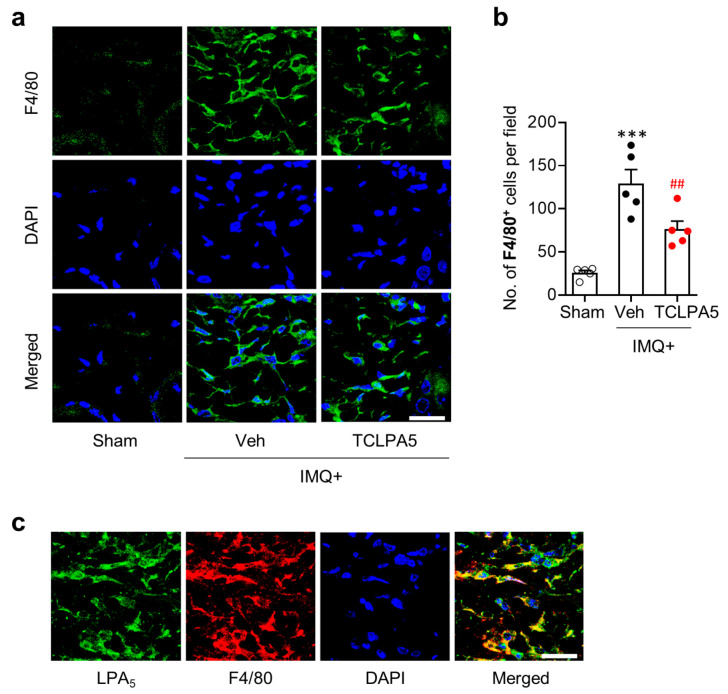
LPA_5_ antagonism reduces macrophage infiltration into psoriasis lesions in IMQ-treated mice. (**a**) Representative photographs of F4/80-labelled skin sections were taken from the dermis of each group. DAPI was used for nuclear staining. (**b**) Quantification of the number of F4/80-immunopositive cells per field (200 µm × 200 µm) was manually performed. n = 5 per group. One-way ANOVA and Newman–Keuls test. *** *p* < 0.001 vs. sham; ^##^ *p* < 0.01 vs. IMQ-treated group (IMQ + Veh). (**c**) Double immunofluorescence labeling of F4/80 and LPA_5_ was performed on skin sections of IMQ-treated mice and representative photographs were provided. Scale bars = 20 µm.

Macrophages are the main cell type for inflammatory responses in psoriasis lesion [28]. They can massively enter into the dermis of psoriasis skin lesion [29]. Thus, we determined whether LPA_5_ could regulate macrophage infiltration in the dermis of psoriasis lesion through immunofluorescence for F4/80. IMQ application significantly increased the number of F4/80-immunopositive cells, while such increase was significantly attenuated upon administration of TCLPA5 at a dose of 2 mg/kg (Figure 3a,b). These data demonstrate that LPA_5_ could promote macrophage infiltration in psoriasis lesions.

In the dermis of psoriasis lesions, LPA_5_ was upregulated (Figure 1b,c). To ascertain if LPA_5_ is localized in infiltrated macrophages, we performed double immunofluorescence for LPA_5_ and F4/80 in IMQ-applied mouse skin. Most of F4/80-immunopositive cells were overlapped with LPA_5_-immunopositive cells in the dermis of psoriasis lesions (Figure 3c), demonstrating that LPA_5_ upregulation in psoriasis lesion mainly occurred in macrophages.

### 3.3. LPA_5_ Regulates NLRP3 Expression in the Dermis of Mice with IMQ-Induced Psoriasis

**Figure 4:**
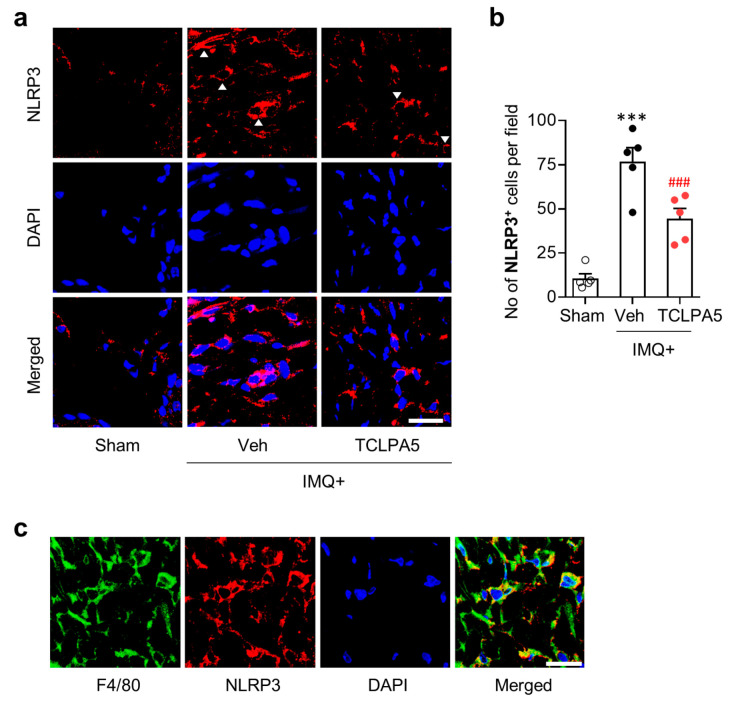
LPA_5_ antagonism attenuates macrophage NLRP3 upregulation in psoriasis lesions of IMQ-treated mice. (**a**) Representative photographs of NLRP3-immunopositive cells in psoriasis lesions were taken from the dermis of each group. Arrowheads indicate NLRP3-immunopositive cells. DAPI was used for nuclear staining. (**b**) Quantification of the number of NLRP3-immunopositive cells per field (200 µm × 200 µm) was manually performed. n = 5 per group. One-way ANOVA and Newman–Keuls test. *** *p* < 0.001 vs. sham; ^###^ *p* < 0.001 vs. IMQ-treated group (IMQ+Veh). (**c**) Double immunofluorescence labeling of F4/80 and NLRP3 was performed on skin sections of IMQ-treated mice and representative photographs were provided. Scale bars = 20 µm.

NLRP3 inflammasome is a key pathogenic event in skin diseases [30]. NLRP3 expression is increased in psoriasis lesion of both human patients and experimental rodent models [31,32]. To address whether LPA_5_ could influence NLRP3 inflammasome activation in psoriasis lesion, we determined NLRP3 expression levels through immunofluorescence. IMQ application significantly increased the number of NLRP3-immunopositive cells mainly in the dermis of psoriasis lesion (Figure 4a,b). TCLPA5 administration significantly reduced the number of NLRP3-immunopositive cells (Figure 4a,b).

Macrophages are the main immune cell type for NLRP3 production in peripheral organs including skin [33,34]. To address whether LPA_5_ could influence psoriasis-induced NLRP3 expression in macrophages, we performed double immunofluorescence for NLRP3 and F4/80 in IMQ-applied mouse skin. Most of F4/80-immunopositive cells were overlapped with NLRP3-immunopositive cells in the dermis (Figure 4c), indicating that NLRP3 upregulation in psoriasis lesion mainly occurred in macrophages. TCLPA5 administration-attenuated immunoreactivities of both F4/80 (Figure 3a,b) and NLRP3 (Figure 4a,b), along with LPA_5_ upregulation in infiltrated macrophages (Figure 3c), collectively suggest that LPA_5_ could regulate NLRP3 inflammasome activation in macrophage to induce inflammatory responses in psoriasis.

### 3.4. LPA/LPA_5_ Signaling Axis Regulates NLRP3 Inflammasome Activation in LPS-Primed BMDMs

**Figure 5:**
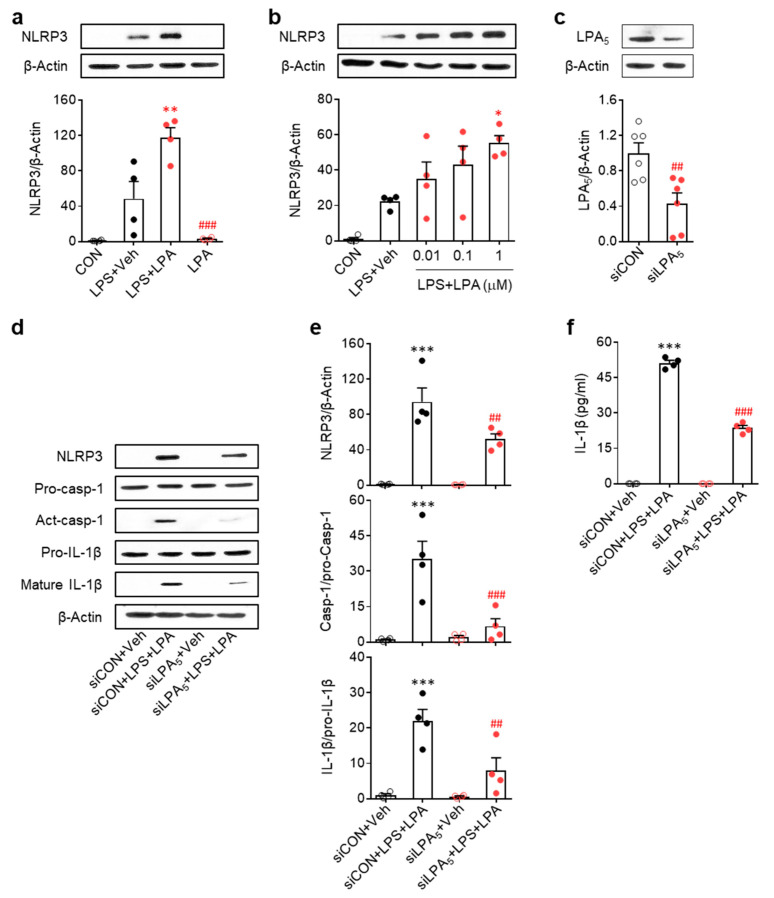
LPA potentiates NLRP3 inflammasome activation in LPS-primed bone marrow-derived macrophages while LPA_5_ knockdown attenuates this activation. (**a**,**b**) Effects of LPA on NLRP3 expression in LPS-primed BMDMs were determined by Western blot analysis. Representative Western blots and quantification of results. One-way ANOVA and Newman–Keuls test. * *p* < 0.05 and ** *p* < 0.01 vs. LPS-treated cells (LPS + Veh); ^###^ *p* < 0.001 vs. LPA and LPS-treated cells (LPS+LPA). LPA was used at 1 µM in (**a**) and at different concentrations (0.01 ~ 1 µM) in (**b**). n = 4 per group. (**c**–**f**) Effects of LPA_5_ knockdown on NLRP3 expression, caspase-1 activation, and IL-1β maturation in LPS-primed BMBM cells were determined. (**c**) Knockdown efficiency of LPA_5_ siRNA. Student’s t test. ^##^ *p* < 0.01 vs. control siRNA (siCON)-transfected cells. n = 6 per group. (**d**–**e**) Representative Western blots (**d**) and quantification of results (**e**). (**f**) ELISA data for IL-1β in culture medium. n = 4 per group. One-way ANOVA and Newman–Keuls test. *** *p* < 0.001 vs. control siRNA-transfected cells (siCON+Veh); ^##^ *p* < 0.01 and ^###^ *p* < 0.001 vs. LPA and LPS-treated cells following transfection with control siRNA (siCON + LPS + LPA).

Because our data highlighted LPA_5_-mediated NLRP3 inflammasome activation in macrophage in vivo, we confirmed its role by modulating expression using siRNA in LPS-primed BMDMs isolated from mice [35,36]. Given data showing that amounts of LPA species were elevated in psoriasis lesions, we first determined whether LPA could increase NLRP3 expression in LPS-primed BMDMs. LPA exposure significantly increased NLRP3 expression in a dose-dependent manner (Figure 5a,b), with 1 µM being the most effective LPA concentration. In addition, LPA exposure significantly induced NLRP3 inflammasome activation as evidenced by NLRP3 upregulation, caspase-1 activation, IL-1β maturation, and IL-1β secretion (Figure 5d–f). Importantly, LPA_5_ knockdown (Figure 5c) significantly attenuated the activation of NLRP3 inflammasome (Figure 5d–f). Taken together, our in vitro results demonstrate that LPA/LPA_5_ signaling axis is associated with NLRP3 inflammasome activation in macrophages, strongly indicating that NLRP3 inflammasome activation is an underlying mechanism of psoriasis governed by LPA_5_ signaling.

## 4. Discussion

The present study revealed a pathogenic role of LPA_5_ signaling in psoriasis using an IMQ-induced mouse model. Amounts of LPA species were elevated and LPA_5_ was upregulated in psoriasis lesions. More importantly, we found that suppressing LPA_5_ activity with a pharmacological antagonist attenuated IMQ-induced psoriasis-like symptoms. It also attenuated macrophage infiltration into psoriasis lesions. In particular, activation of LPA_5_ signaling was found to upregulate macrophages NLRP3 expression in psoriasis lesions. Additional in vitro studies revealed that LPA could activate NLRP3 inflammasome in LPS-primed macrophages through LPA_5_. These data provide evidence that LPA_5_ signaling plays a critical role in psoriasis through its mechanistic role for regulation of macrophage NLRP3 inflammasome activation.

Under physiological conditions, LPA is present at higher concentrations in blood than in other tissues [37]. However, it is important to note that local LPA production is more likely to be associated with disease pathology than circulating LPA [38]. This notion could be supported by a previous study on skin itching in mice by local injection of 1-oleoyl-LPA into the cheek [16]. In that study, LPA_5_ was suggested as a possible mediator through in vitro studies using sensory neurons of the dorsal root ganglion. In the current study, amounts of LPA species had more dramatic elevation in local skin lesions than in plasma. Such increase of local LPA level could be important for disease development in mice with IMQ-induced psoriasis. Although we did not determine direct effects of LPA itself on psoriasis-like symptoms, our results clearly suggested that LPA_5_ was a pathogenic factor for psoriasis based on LPA_5_ upregulation in psoriasis lesions and attenuated psoriasis-like symptoms in IMQ-treated mice by its antagonism. Therefore, increased ligand levels could influence psoriasis pathogenesis through LPA_5_.

Macrophage modulation has become a new strategy to prevent inflammatory skin diseases [6,39]. Dermal infiltration of macrophages and their classical activation towards inflammatory phenotypes are well-reported in psoriasis lesions [29]. Therefore, attenuating infiltration of macrophages and their proinflammatory polarization is an appealing therapeutic approach to treat psoriasis [28]. Importantly, macrophages are the major cell type for the inflammatory responses in psoriasis lesions of human patients [40]. Chlodronate liposome, a selective macrophage depleting agent, can significantly attenuate psoriasis symptoms [41], indicating that macrophage is a promising therapeutic target in psoriasis. Recent reports have suggested that LPA signaling could be an important regulator of macrophage biology since it can regulate the conversion of monocytes to macrophages, promote their activation, and increase M1 polarization [41,42,43,44]. These previous studies strongly indicate that LPA signaling could modulate activation and infiltration of macrophages in psoriasis lesion to trigger inflammatory cascades. Moreover, previous studies showing gene expression levels of LPA receptors have demonstrated that LPA_5_ is predominantly expressed in alveolar macrophages [45] and tumor-associated macrophages [15]. Indeed, we found that suppressing LPA_5_ activity could attenuate macrophage infiltration into the dermis of psoriasis lesion, implicating that a pathogenic role of LPA_5_ could be closely linked to macrophage infiltration into psoriasis legions. Moreover, we found that LPA_5_ was upregulated in these infiltrated macrophages, implicating that LPA_5_ could regulate functions of macrophages in lesion areas.

Although we focused on the impact of LPA_5_ on macrophage modulation in psoriasis, LPA_5_ could also contribute to psoriasis lesions by modulating functions of other psoriasis-associated cell types, such as keratinocyte [46,47]. Topical LPA application can increase keratinocytes proliferation and epidermal thickness [48] and ameliorate skin barrier function through LPA_1_/LPA_5_ [17]. Sumitomo et al. [17] have examined filaggrin expression to assess keratinocyte differentiation and skin barrier function because filaggrin was associated with skin diseases such as dry skin and atopic dermatitis. Even though loss-of-function mutations in the gene of filaggrin are not associated with psoriasis [38], it is sure that LPA_5_-mediated LPA signaling influences keratinocyte biology [17] and keratinocytes are the major cell type to contribute to psoriasis lesions [46,47]. Therefore, roles of LPA_5_ in psoriasis could be additionally associated with regulation of keratinocyte biology. Besides keratinocyte biology, LPA_5_ might be also able to affect T cell biology in psoriasis. Infiltration of T cells in the lesion sites is considered as a critical pathogenic event in psoriasis [49]. In fact, T cells depletion therapy has been well accepted in patients with psoriasis and inhibition of IL-17 producing T cells has exerted potential clinical efficacies to treat psoriasis [50,51]. Infiltrated T cells in psoriasis lesions are associated with production of cytokines and chemokines which further attract other immune cells and aggravate the inflammatory cascades in psoriasis [52]. LPA_5_ is highly expressed on T cells [12]. In addition, we demonstrated that TCLPA5 administration reduced mRNA expression levels of IL-17 that can be produced mainly by T cells [53]. Therefore, it remains possible that LPA_5_ may play important roles in psoriasis by regulating T cell biology.

NLRP3 inflammasome has been considered as an important inflammatory mediator in diverse diseases, leading to validation of its importance as a therapeutic target of inflammatory diseases [54]. In general, NLRP3 inflammasome activation in macrophages requires two signals [55]. The first signal (priming signal) is mediated by toll-like receptor ligands such as LPS or cytokines such as TNF-α. It activates NF-κB, resulting in upregulation of NLRP3 and/or pro-IL-1β. The second signal (activation signal) is mediated by pathogen-associated molecular patterns or damage associated molecular patterns stimulations such as ATP, resulting in promotion of NLRP3 inflammasome assembly and caspase-1-mediated IL-1β maturation. Numerous efforts have been made to reveal endogenous/exogenous stimuli [56] and G protein-coupled receptors [57] as regulators of NLRP3 inflammasome activation. In the current in vitro study, LPA was first demonstrated to be able to activate NLRP3 inflammasome in macrophages. Although LPA itself did not induce NLRP3 upregulation, NLRP3 expression was further upregulated by LPA in LPS-primed macrophages. LPA also induced caspase-1 activation, IL-1β maturation, and IL-1β secretion in LPS-primed macrophages. These results indicate that LPA could activate NLRP3 inflammasome in primed macrophages. In particular, LPA_5_ was found to be able to regulate this LPA-driven NLRP3 inflammasome activation in these cells. More importantly, our in vivo studies demonstrated that amounts of LPA species in psoriasis lesions were elevated and that suppressing LPA_5_ activity could attenuate NLRP3 upregulation in psoriasis lesions, particularly in infiltrated macrophages. Therefore, activation of LPA_5_ signaling might contribute to skin injury in psoriasis, in which NLRP3 inflammasome activation could be an underlying mechanism. This NLRP3-relevant mechanistic notion could be supported by previous reports showing that NLRP3 expression is upregulated in human psoriasis biopsy [32] and that genetic deletion of NLRP3 can significantly ameliorate skin thickening in mice with IMQ-induced psoriasis [31].

Medically relevant roles of receptor-mediated LPA signaling in psoriasis have emerged, particularly after a clinical trial for an LPA_1_ antagonist in psoriasis. Based on current findings, LPA_5_ could be an additional LPA receptor type with medically relevant roles in psoriasis, further implicating that psoriasis could be therapeutically treated through LPA_5_ antagonism. Moreover, in view of the regulatory role of LPA_5_ in NLRP3 inflammasome activation, targeting LPA_5_ could be a tempting strategy to treat a variety of NLRP3 inflammasome-mediated diseases.

## Figures and Tables

**Table 1:** Topical application of imiquimod increases LPA amount in mice.

LPA Species	Skin		Plasma	
	Fold Changes	*p* Value	Fold Changes	*p* Value
16:0	1.26	0.376	0.90	0.020
16:1	4.34	0.000	0.40	0.000
16:2	5.09	0.000	N.D.	
16:3	19.93	0.000	0.07	0.024
17:0	7.64	0.002	0.75	0.000
17:1	2.14	0.026	N.D.	
17:2	3.48	0.001	2.29	0.026
18:0	18.39	0.000	1.12	0.001
18:1	2.46	0.335	1.20	0.039
18:2	2.11	0.000	0.73	0.003
18:3	2.83	0.004	N.D.	
18:4	11.56	0.000	N.D.	
18:5	N.D.		2.02	0.000
19:0	9.49	0.005	N.D.	
20:0	7.46	0.316	N.D.	
20:1	0.56	0.020	N.D.	
20:2	75.13	0.279	N.D.	
21:0	N.D.		1.44	0.015
21:1	20.17	0.068	1.40	0.024
22:6	1.00	0.994	N.D.	

The amount of different LPA species in the skin tissue lysate and in plasma of sham and IMQ-treated mouse was measured at 7 days after IMQ treatment using LC/MS. n = 10 for sham and n = 9 for IMQ. Two-tailed t-test. N.D., not detected.
